# Dual Energy CT Physics—A Primer for the Emergency Radiologist

**DOI:** 10.3389/fradi.2022.820430

**Published:** 2022-02-24

**Authors:** Devang Odedra, Sabarish Narayanasamy, Sandra Sabongui, Sarv Priya, Satheesh Krishna, Adnan Sheikh

**Affiliations:** ^1^Department of Radiology, University of Toronto, Toronto, ON, Canada; ^2^Department of Radiology, Carver College of Medicine, The University of Iowa, Iowa City, IA, United States; ^3^Keenan Research Centre for Biomedical Science, St Michael's Hospital, Toronto, ON, Canada; ^4^Department of Medical Imaging, Mount Sinai Hospital, and Women's College Hospital, University Health Network, University of Toronto, Toronto, ON, Canada; ^5^Department of Radiology, The University of British Columbia, Vancouver, BC, Canada

**Keywords:** dual energy (CT), physics, emergency radiology, material decomposition, CT

## Abstract

Dual energy CT (DECT) refers to the acquisition of CT images at two energy spectra and can provide information about tissue composition beyond that obtainable by conventional CT. The attenuation of a photon beam varies depends on the atomic number and density of the attenuating material and the energy of the incoming photon beam. This differential attenuation of the beam at varying energy levels forms the basis of DECT imaging and enables separation of materials with different atomic numbers but similar CT attenuation. DECT can be used to detect and quantify materials like iodine, calcium, or uric acid. Several post-processing techniques are available to generate virtual non-contrast images, iodine maps, virtual mono-chromatic images, Mixed or weighted images and material specific images. Although initially the concept of dual energy CT was introduced in 1970, it is only over the past two decades that it has been extensively used in clinical practice owing to advances in CT hardware and post-processing capabilities. There are numerous applications of DECT in Emergency radiology including stroke imaging to differentiate intracranial hemorrhage and contrast staining, diagnosis of pulmonary embolism, characterization of incidentally detected renal and adrenal lesions, to reduce beam and metal hardening artifacts, in identification of uric acid renal stones and in the diagnosis of gout. This review article aims to provide the emergency radiologist with an overview of the physics and basic principles of dual energy CT. In addition, we discuss the types of DECT acquisition and post processing techniques including newer advances such as photon-counting CT followed by a brief discussion on the applications of DECT in Emergency radiology.

## Introduction

Multidetector Computed Tomography (MDCT) has revolutionized diagnostic radiology and has become the workhorse of medical imaging, especially in the emergency department (1). Conventionally, MDCT has been performed with a “single energy” where a single polychromatic x-ray beam (typically generated by a 100–120 kVp tube voltage) is generated by a single source and detected by a single detector array. Dual energy and multi-energy CT involve acquiring images at two or more energy spectra at different kilovoltage peaks (kVp). Acquisition of images at two energy levels allows for differentiation of materials with different atomic numbers which may have the same CT attenuation. Although the concept of dual energy CT acquisition was initially introduced in the early 1970s, it has been only over the past two decades that it has been used widely in routine practice. DECT can be used to generate virtual monochromatic images, material density images, virtual unenhanced images, calcium subtracted images, Iodine maps and can be used to reduce artifacts such as beam hardening and metallic artifacts. The purpose of this article is to discuss the basic principles of DECT, considerations for image acquisition and techniques, and a brief overview of clinical applications of DECT particularly in emergency radiology. Specific clinical applications of DECT are available elsewhere in this issue.

## Basic Physics

In conventional CT, the image is generated by the attenuation of the photon beam by various materials encountered in its path such as muscle, fat, fluid, bone, metal, or intravenous iodinated contrast material (2). The attenuation of the x-ray beam is a function of the energy of the x-ray beam and the density and atomic number of the attenuating material. Two main phenomena govern the attenuation in the range of energies encountered in medical imaging: photoelectric effect and Compton scatter. The photoelectric effect refers to removal of an electron from an inner shell of the atom by the incident photon. Each material has a characteristic k-shell energy level, which is the minimum energy required by the incoming photon to remove an inner-shell electron. At mean energies just above the k-shell energy level, the attenuation of the incoming photon beam is the highest with subsequent tapering at higher energy levels. This is referred as “k-edge” or “k-absorption edge” of the material.

In conventional CT with a single energy spectrum, many materials with different atomic numbers may have similar attenuation values. Although calcium and iodine have different atomic numbers, they can demonstrate similar CT attenuation on conventional CT images and it is therefore often difficult to differentiate between atherosclerotic calcification and intraluminal contrast on a CT angiogram, or between a renal stone and excreted contrast into the renal pelvis (3). The core principle of DECT lies in the fact that the absorption of the incoming photon varies as a function of energy. When exposed to a single-energy beam, two different materials may have the same attenuation value. However, when exposed to a second beam of a different energy closer to one of the materials' k-edge, the materials may demonstrate different attenuation values. This differences in attenuation at two different energies can be utilized to identify and characterize the individual materials (4). For e.g., at 120 kVp single-energy CT, both calcium in bone and high-density iodinated contrast material may have very similar attenuation values. However, when exposed to a lower energy X-ray beam generated by a 70 kVp tube voltage (which is closer to the k-edge of iodine), the iodinated contrast would exhibit a higher attenuation than calcium, thus enabling separation of the two materials. The two kVp settings commonly used in DECT in current practice are 80 kVp and 140 kVp (5, 6).

## Dect Acquisition Techniques

**Figure 1:**
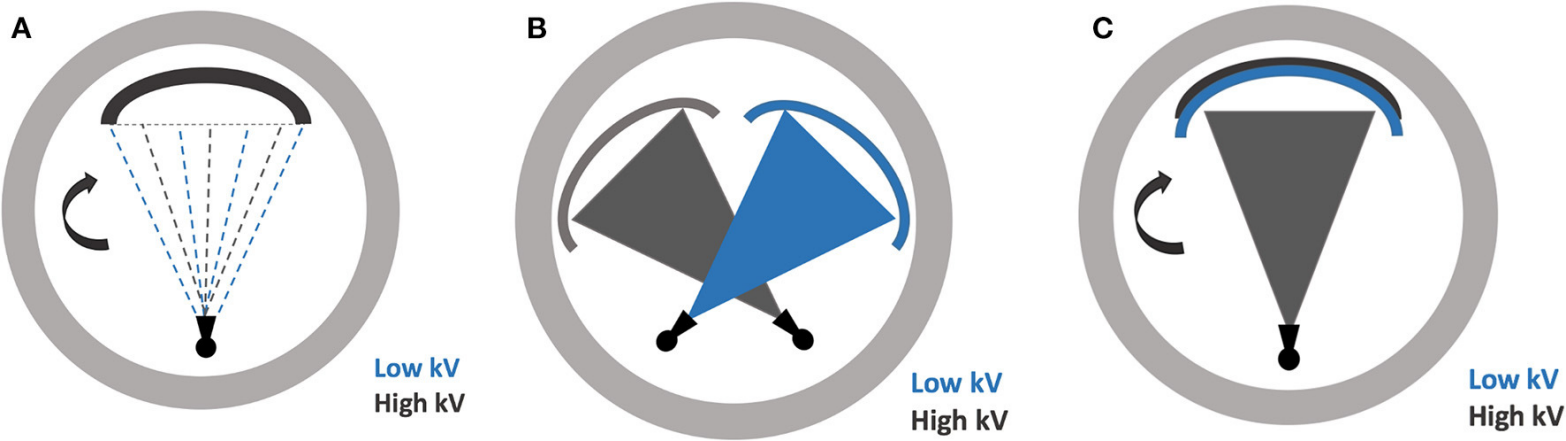
Illustrations demonstrating the various dual-energy CT scanning techniques **(A)** rapid energy switching **(B)** dual source scanning **(C)** multilayer detector.

DECT requires two different datasets acquired at two energy levels. Several hardware strategies have been developed by different vendors for image acquisition in DECT (Figure 1).

### Dual-Scanning Technique

The dual-scanning technique is essentially a repeated scan of the same anatomical region at two different energy settings, amounting to a “dual scan”. This can be achieved by sequentially repeating either the entire target volume or each axial slice acquisition. The major advantage of this technique is that it can be easily implemented on existing equipment without any additional upgrades or replacements. A major disadvantage, however, is the misregistration due to movement in between the acquisitions (4, 6, 7).

### Rapid Energy Switching

In this type of technique, the x-ray source can rapidly switch between low and high energy beam, hence shortening the length of scan and minimizing any motion artifacts. However, the tube current is usually unchanged throughout the kV-switching process with proportional exposure allocated to each energy. A major disadvantage of this method is the need for a specialized hardware, as well as the overlap in energy spectrum between the two energy settings, given the rapid switching (4, 6, 7).

### Dual-Source Technique

In this technique, there are two x-ray sources, mounted orthogonally to each other. These operate independently, allowing optimal control of scan parameters for each source. Additionally, the sources operate simultaneously, offering exceptional temporal resolution as only a quarter rotation is required for each detector for image reconstruction. Two independent sources also allow no overlap in the energy spectra, which improves contrast-to-noise ratio. The hardware is however expensive and intensive. There is also a need for scatter correction algorithms as scatter from one detector can confound the image detected by the other (4, 6, 7).

### Multilayer Detector

Rather than exposing the target with two separate beams of energy levels, a specialized multilayered or “sandwich” scintillation detector allows for differentiating the energy levels at the detector level. The inner layer of detectors collect data from the low energy photons and the outer layer of detectors collect data from the high energy photons. As such, this system always operates in the dual-energy mode, allowing retrospective dual-energy analysis for any study. The low and high energy is acquired simultaneously, with no impact on the scan times or motion artifact. However, the system requires specialized equipment. Additionally, the lack of control over the scan parameters for each energy compromises the image quality. This is partly mitigated by the utilization of a variable detector thicknesses (4, 6, 7).

### Photon Counting CT

Photon counting CT (PCT) is a recent development in CT technology and has shown tremendous progress in the last few years. The mechanism of the photon counting CT differs substantially from a conventional CT. It utilizes a single energy x-ray source with a single thick layer of energy resolving detectors. The detectors in PCT do not use scintillators and use direct conversion technology to convert the x-rays directly into charged particles. The detectors count the number of incoming attenuated photons individually and separate them into different photon bins based on their energy levels (8). This allows for multi energy image acquisition from a single x-ray energy source. The major advantages of photon counting CTs include ability to obtain images with a high contrast to noise ratio and high spatial resolution, decrease electronic noise, potential for radiation dose reduction and reduction of beam hardening and metallic artifacts (9).

## Types of Images Generated and Post-Processing Techniques

Several image output options are available for DECT, depending on the purpose of the study. While two polychromatic beams with low and high average kVp are used for image acquisition, mathematical post-processing algorithms and modeling allows for reconstruction of virtual monoenergetic images (VMI) at any energy level between the two levels. This unique advantage of DECT allows the radiologist to optimize the images based on the clinical interest. For e.g., Low energy virtual monochromatic image are used to maximize the visualization of iodinated contrast and improve the contrast to noise ratio. VMC images created at 50 keV have been reported to demonstrate peak contrast to noise ratio (10). On the other hand, high-energy > 90 keV reconstruction images minimizes artifact from metallic hardware (11, 12). Additionally, a mixed or weighted image is generated from the average of the two datasets, typically a combination of 50–60% low energy and 40–50% high energy images (typically at 70 keV), which simulates the conventional single energy image. These images are typically used for routine diagnostic interpretation.

One of the true benefits of DECT lies in its ability for material characterization. Material specific images can be generated from high and low energy projection data and can show the distribution of a particular material on the CT images (7). This can be accomplished by using either a two-material decomposition or three material decomposition algorithms. Post-processing algorithms can also generate images with the target material subtracted (e.g., virtual unenhanced study from iodine subtraction), target material only (e.g., iodine map) or a combination (e.g., virtual unenhanced with superimposed iodine map). Similar analysis can also be performed for bone (e.g., calcium-subtracted angiographic images to minimize artifact from atherosclerotic plaque). In addition, material specific images can also provide quantitative information about the target material (e.g., quantification of calcium distribution in vessels or to provide quantitative assessment of hepatic steatosis).

## Radiation Dose

In its early days, DECT was associated with a significant increase in radiation dose, as it essentially amounted to scanning the patient twice. However, improvements in DECT detectors have led to significantly shorter scanning times, as well as multi-layered detectors for allowing differential data acquisition for multiple energy beams. Advances in x-ray source technology have allowed for tools such as fast kV switching and software innovations have resulted in advanced post-processing and iterative reconstruction algorithms, allowing for improved image quality at a lower dose (13). Numerous recent studies have shown that radiation dose in DECT is comparable, if not less, than conventional single energy CT (14–16). Dose reduction is especially relevant in coronary CT angiography where it has been shown that radiation dose associated with dual energy cardiac CT is significantly lower than conventional CT while providing equivalent diagnostic information (15, 17).

One of the ways DECT minimizes radiation dose is by obviating the need for unenhanced images, which can readily be extracted from contrast-enhanced examination by identifying and subtracting the iodine from the images (18). This leads to reduction in radiation dose in multi-phasic examinations such as CT urography, post-EVAR CT angiography and multiphasic abdominal CT. What was once double the radiation dose, is now nearly comparable to a single-energy scan (19). Furthermore total dataset in DECT is split between the two energy beams in dual-source and fast-KV techniques, rather than duplicating two full single-energy scans (4). The superior contrast to noise ratio of DECT also enables to reduce the radiation dose and also to decrease the dose and/or rate of contrast volumes administered compared to single energy CT without a detrimental effect on image quality (20).

## Applications in Emergency Radiology

There have been numerous applications of DECT in radiology and the list is growing with time. From head to toe, DECT can aid in the detection, diagnosis and management of many conditions, including in an acute setting (21–24). A brief overview of applications in emergency radiology is presented below and a more thorough review of these applications will follow in other articles in this issue.

**Figure 2:**
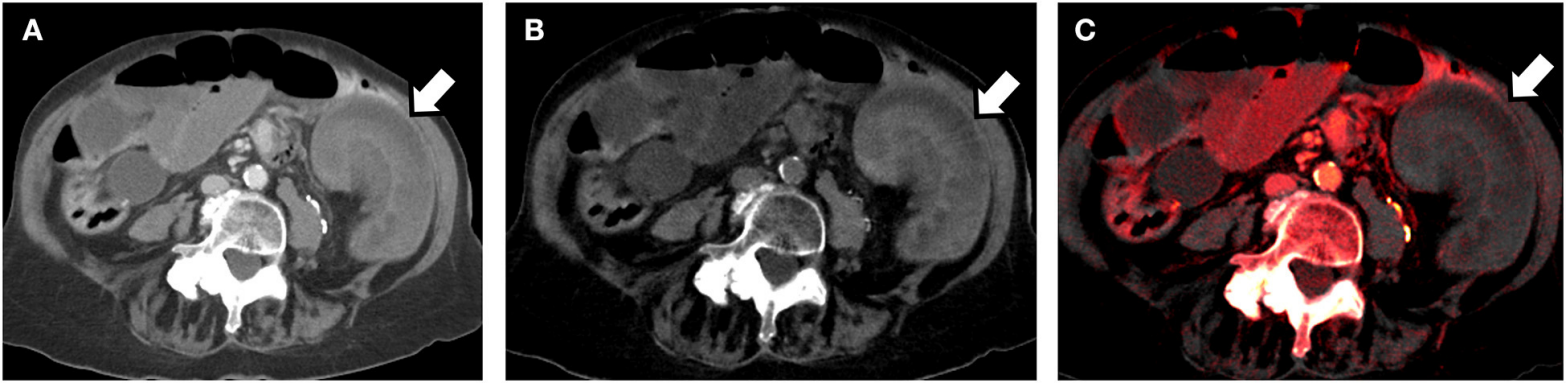
80-year old man presenting to the Emergency Department with acute abdominal pain. Dual energy CT (using a dual-source scanner) in the portal venous phase was performed. **(A)** Axial DECT 70 keV image (simulated 120 kVp image) shows a loop of small bowel in the left side of the abdomen which appears to be thickened (arrow). It is unclear if the bowel is normally enhancing or not. **(B)** Axial virtual unenhanced image reconstructed using the dual-energy dataset shows that the bowel loop is hyperattenuating. This would imply intramural hemorrhage. **(C)** Axial iodine color overlay images reconstructed from the dual energy data set, show lack of color in the bowel wall implying lack of iodine and lack of enhancement. Bowel ischemia was confirmed at laparotomy.

DECT can perform iodine mapping and demonstrate differential enhancement of brain parenchyma in acute stoke and also to differentiate between venous thrombosis from iodine flux artifacts (24). Material specific iodine images have also been used to differentiate intraparenchymal hemorrhage and contrast staining in stroke (25). VNC images can be reconstructed from contrast enhanced acquisitions and approximates true non-contrast images. This can reduce the number of acquisitions and radiation exposure and has been used in the imaging of acute aortic syndromes for diagnosis of intramural hematomas (26). VNC images have also shown to reduce the need for additional studies in characterizing incidentally detected adrenal nodules (27). Iodine specific images have been used to determine the absence of enhancement in hyperdense renal cysts incidentally detected on single phase contrast studies thereby differentiating it from enhancing hyperdense solid nodules (28). DECT can also be used to demonstrate bone marrow edema in occult fractures and absence of bowel wall enhancement in acute ischemia (Figure 2) (29, 30).

Iodine mapping is also useful in diagnosis of acute pulmonary embolism. High energy virtual mono-chromatic images can be used to reduce beam hardening. The ability to characterize material plays an integral role in genitourinary imaging where identification of certain types of renal stones can guide management. Breakdown of enhancement characteristics enhances diagnosis of acute abdomen by demonstrating ischemic bowel (31). Calcium subtraction can better characterize the vessel patency in cases of acute limb ischemia. There are many more applications of DECT in non-emergent setting, with excellent reviews published previously (4, 32, 33).

## Conclusion

While the concept of DECT is certainly not recent, technological advances in hardware and software have made it more applicable and feasible in recent times. There are several established applications of DECT that aid in detection, diagnosis, and management of several acute and non-acute pathologies. The gamut of its applications continues to rise, with more applications on the horizon. As more institutions adopt newer generation scanners and technologies with time, DECT will emerge as a valuable player in the radiologist's toolkit.

## Author Contributions

DO and SN wrote the initial manuscript. SP and SS were involved in proof reading, revision, and preparation of figures. AS and SK devised the project, conceptual outline, and revised the final version of the manuscript. All authors approved the submission of the final manuscript.

## Conflict of Interest

The authors declare that the research was conducted in the absence of any commercial or financial relationships that could be construed as a potential conflict of interest.

## Publisher's Note

All claims expressed in this article are solely those of the authors and do not necessarily represent those of their affiliated organizations, or those of the publisher, the editors and the reviewers. Any product that may be evaluated in this article, or claim that may be made by its manufacturer, is not guaranteed or endorsed by the publisher.
